# 

**Published:** 1873-09

**Authors:** 


### CONTENTS:

### ORIGINAL COMMUNICATIONS.

Article I.—Proceedings of Fifth Annual Meeting of the Southern Dental Association...... 19[illegible]

Article II.—The Southern Dental Association and the Dental Colleges...................... 21[illegible]

Article III.—Physiology of the Dental Structures. By S. P. Cutler, D. D. S., M. D.... .. 2[illegible]

### SELECTED ARTICLES.

Article IV.—A New Method of Producing Local Anæsthesia. By A. Horvath, M D....... 23[illegible]

Article V.—Bromide of Potassium and the Diseases of Dentition. By C. G. Polk, M. D. 23[illegible]

Article VI.—Collis' Method of Operating for Hare-Lip. By T. A. McGraw, M. D......... 23[illegible]

### EDITORIAL, ETC.

Separating Teeth........................................................ 234

### MONTHLY SUMMARY.

Chloral as an Application to Fetid Ulcers............................ 237
Death from Methylene Ether............................................ 238
Cholera................................................................ 238
Ingrowing Toe Nails.................................................... 239
Detection of the Substitution of Carbolic Acid for Creasote ............ 239
Effects of the Suppression of Perspiration.............................. 240

